# Sclerotherapy as an alternative treatment for complex, refractory seromas

**DOI:** 10.1093/jscr/rjab224

**Published:** 2021-08-24

**Authors:** Nicole C Episalla, Susan Orra, Cara K Black, Paige K Dekker, Kevin G Kim, John T Cardella, Karen K Evans

**Affiliations:** Department of Plastic and Reconstructive Surgery, Medstar Georgetown University Hospital, Washington, DC 20007, USA; Department of Plastic and Reconstructive Surgery, Medstar Georgetown University Hospital, Washington, DC 20007, USA; Department of Plastic and Reconstructive Surgery, Stanford University Medical Center, Palo Alto, CA 94304, USA; Department of Plastic and Reconstructive Surgery, Medstar Georgetown University Hospital, Washington, DC 20007, USA; Department of Plastic and Reconstructive Surgery, Medstar Georgetown University Hospital, Washington, DC 20007, USA; Department of Interventional Radiology, Medstar Georgetown University Hospital, Washington, DC 20007, USA; Department of Plastic and Reconstructive Surgery, Medstar Georgetown University Hospital, Washington, DC 20007, USA

## Abstract

Traditional therapy for seromas often entails compression, aspiration, drainage, or surgical excision and re-closure; however, more complex, treatment-refractory seromas may require additional treatment. Sclerotherapy has been well documented in the treatment of simple pleural effusions, vascular malformations, lymphoceles and seromas. However, little evidence is available on the efficacy of sclerotherapy in complex, treatment-refractory seromas that develop post-operatively in patients with complex medical histories. We present a case series highlighting the use of sclerotherapy by interventional radiology as an alternative or adjunctive treatment method for chronic, high-volume post-operative seromas recalcitrant to multiple attempts of traditional treatment. At long-term follow-up, the seromas resolved after a maximum of four rounds of sclerotherapy with various combinations of known sclerosants. Highly complex cases of large, chronic seromas may be refractory to conservative modalities and re-closure. Sclerotherapy can be considered an alternative method or adjunctive treatment for chronic, recalcitrant post-operative seromas.

## INTRODUCTION

Post-operative seroma is a common complication that occurs in dead spaces left behind by surgical dissection or trauma [1, 2]. Fluid from transected blood and lymphatic vessels as well as inflammatory fluid accumulate in the dead space, creating a fluid collection [1–4]. Without intervention, the fluid collection can increase in size and eventually develop a fibrous pseudocapsule, potentially resulting in discomfort, contour deformity, compression of surrounding structures and even abscess [2, 4].

Strategies to prevent seroma formation have been well established by Janis *et al* [2]. Prevention strategies include the use of closed-suction drains with high pressure until drain output is minimal, ultrasonic or sharp dissection instead of cautery, progressive tension sutures, ligating vessels with clips or suture, immobilization, and use of fibrin, talc or thrombin [2]. Despite these measures, seromas may still form. Although most seromas are successfully treated conservatively with serial aspiration, some patients require re-operation with excision of the pseudocapsule, drain placement, and re-closure. More complex, refractory seromas may require additional treatment with repeated percutaneous drainage, further surgical procedures and sclerotherapy [1, 3]. Sclerotherapy is a procedure that utilizes a chemical agent (sclerosant) to irritate the inner lining of a seroma, inducing an inflammatory reaction resulting in fibrosis and closure of the fluid cavity [1].

Sclerotherapy has been well documented in the treatment of simple pleural effusions, vascular malformations, lymphoceles and seromas [2, 4–7]. However, little literature is available on the efficacy of sclerotherapy in complex, treatment-refractory seromas that develop post-operatively in patients with complex medical histories. We present a case series demonstrating the success of sclerotherapy in treating cases of highly complex, treatment-refractory seromas.

Two patients with seromas in the thigh and one patient with a seroma in the retroperitoneum were included in this study. All patients had high-volume seromas refractory to compression, aspiration, drainage and surgical excision and re-closure, and thus received treatment with sclerotherapy. Sclerosants used in this study include doxycycline, povidone-iodine, 3% sodium tetradecyl sulfate (STS) and absolute ethanol. All treatment was provided from December 2017 to April 2018. Review of patient records was permitted under IRB 2018-173 (Plastic Reconstructive Surgery Outcomes Registry).

## CASES

### Case 1: persistent seroma following necrotizing faciitis

A 30-year-old male with a history of hypertension, anemia, hyperlipidemia, tobacco use and chronic kidney disease secondary to focal sclerosing glomerulonephritis intermittently on immunosuppressants developed necrotizing fasciitis of the right medial thigh requiring multiple debridements. He later presented with a large seroma in the surgical site as well as sepsis 1 month post-operatively. The patient underwent debridement of the seroma twice, and the wound was closed over a drain. Initial cultures of the wound grew methicillin-resistant *Staphylococcus aureus*, and the appropriate antibiotics were given. The patient had persistently high drain output post-operatively and consequently underwent sclerotherapy on post-operative Day 4 with doxycycline and post-operative Day 5 with povidone-iodine.

Ten days later, the patient continued to have high serous drain output and developed induration and fluctuance at the previous incision site. The seroma was 9.4 × 3.3 × 2.3 cm by computed tomography (CT). The wound now grew multi-drug resistant *Pseudomonas aeruginosa* and appropriate antibiotics were given. The patient underwent sclerotherapy with STS but continued to have high drain output. Lymphocele was ruled out at this time with nuclear magnetic lymphoscintigraphy. The patient then underwent a third round of sclerotherapy with STS with a resultant decrease in drain output. The patient had no evidence of fluid collection on ultrasound 5 days after the last round of sclerotherapy. The patient had no clinical evidence of seroma recurrence 2 years later.

### Case 2: persistent seroma following resection of leiomyosarcoma

A 62-year-old female with a history of prediabetes, hyperlipidemia, hypothyroidism, breast cancer status post radical mastectomy and chemotherapy, and deep vein thrombosis of the right internal jugular vein underwent resection of a leiomyosarcoma with positive margins from the right proximal medial thigh. She later presented with an enlarging mass believed to be a seroma in the surgical site. The seroma was aspirated, but the mass continued to expand thereafter. T2 magnetic resonance imaging at this time revealed a 15.0 × 15.0 cm seroma with attachment to underlying muscle. The patient then underwent debridement of the seroma and resection of residual disease with negative margins. The resultant defect was reconstructed with a sartorius muscle flap and an anteromedial thigh perforator V-Y flap.

At outpatient follow-up 10 days post-operatively, the patient had drainage of 200–300 cc/day of clear, serous fluid, which persisted at 4-week follow-up. The patient therefore underwent sclerotherapy with doxycycline but continued to have swelling and drainage of 150–200 cc/day, requiring additional sclerotherapy with STS 4 weeks later. The patient had no evidence of seroma recurrence 2 years later.

### Case 3: persistent seroma following anterior approach spinal fusion

A 68-year-old obese male with a history of hypertension, prostatectomy and a motor vehicle accident (MVA) 21 years ago requiring multiple surgeries underwent an anterior approach lumbar spinal fusion for persistent back pain from the MVA. The patient presented with a CT confirmed seroma measuring 7.0 × 9.0 × 12.5 cm retroperitoneally along the anterior margin of the psoas 7 months post-operatively. The patient underwent CT-guided aspiration. The seroma persisted for 3 months thereafter, and the patient again underwent image-guided aspiration and drain placement. The drain was found to be partially outside of the seroma 2 weeks after the second aspiration and was therefore removed.

Two weeks after drain removal, the seroma increased in size and became infected. The abscess was drained and a new drain was placed. The abscess grew *P. aeruginosa*, *Enterococcus faecalis* and *Eikenella* species and was treated with the appropriate antibiotics. Drain output decreased to ~20–40 cc per day. Enteric fistula was ruled out at this time. The patient then underwent three rounds of sclerotherapy with STS approximately every 2 weeks. The patient underwent a fourth round of sclerotherapy with povidone-iodine. CT 1 week later revealed resolution of the fluid collection, and the drain was removed. The seroma resolved completely at 6 week follow-up and had no recurrence 2 years later.

## DISCUSSION

Sclerotherapy is a useful alternative method of treating refractory, high-volume seromas in patients with complex medical histories. All patients in this study had large, chronic seromas that failed multiple attempts at traditional treatment. They also had multiple comorbidities that likely interfered with wound healing, such as obesity, smoking history and cancer history. Some patients had complicated surgical histories. Furthermore, two cases described in this study had infected seromas. Despite the complexity of these cases, all seromas resolved after a maximum of four rounds of sclerotherapy.

All patients were initially treated with widely accepted, clinically proven methods of therapy including compression bandaging [8–10]. Aspiration has also been proven clinically effective, especially in small seromas of <50 ml [11]. Nickerson *et al.* [11] assert that seromas that contain >50 ml of fluid or are refractory to aspiration may require additional forms of treatment. Balch *et al*. [12] have found success in drain placement in seromas that persist for 2–3 weeks after aspiration. Surgical debridement and excision of seroma pseudocapsule is also widely accepted in the literature as one of the most effective, albeit invasive, treatments for seromas [13–15]. Carlson *et al*. [13] treated 24 seromas with surgical debridement without recurrence or post-operative infection. Although highly effective in most seromas, these therapies were unsuccessful in the described cases likely due to the size and chronic nature of the patients’ seromas, as well as the complexity of the patients’ medical and surgical histories.

Two successfully treated seromas in this series were infected and treated with antibiotics, raising the question of the impact infection has on treatment. The review of current literature reveals a consensus opinion that an inflammatory process must be generated to cause fibrosis of the seroma cavity. Since infection is known to result in an inflammatory reaction and subsequent fibrosis, the question arises whether the presence of an intra-cavitary infection may also facilitate fibrosis thereby decreasing the number of sclerotherapy rounds required to achieve resolution. Luria *et al*. [16] suggested that infected seromas require longer duration of treatment, despite the inflammatory reaction, due to increased fluid accumulation inside of the seroma cavity.

Although this case series is a limited example, it provides evidence that sclerotherapy is efficacious in treating highly complex seromas that are recalcitrant to more traditional treatment modalities. Further investigation is required to reinforce these findings. Early use of sclerotherapy, which is less invasive and can be performed in clinic or as outpatient surgery, can limit patient exposure to invasive, painful procedures such as repeated aspiration and surgical debridement. In addition, few complications and low occurrence of infection have been reported after sclerotherapy in a systematic review by Sood *et al*. [1].

Although many seromas resolve with traditional therapies such as compression, aspiration, drainage or surgical debridement, some may persist despite utilization of these treatment options. In these cases, surgeons should recognize sclerotherapy as an effective treatment alternative.
